# Methodological approaches for conducting follow-up research with clinical trial participants: a scoping review and expert interviews

**DOI:** 10.1186/s13063-021-05866-6

**Published:** 2021-12-27

**Authors:** Aita Signorell, Jasmina Saric, Christian Appenzeller-Herzog, Hannah Ewald, Christian Burri, Martin Goetz, Jana Gerold

**Affiliations:** 1 Swiss Tropical and Public Health Institute, P.O. Box, CH-4002, Basel, Switzerland; 2 University of Basel, P.O. Box, CH-4003, Basel, Switzerland; 3 University Medical Library Basel, University of Basel, P.O. Box, CH-4003, Basel, Switzerland; 4 Federal Office of Public Health, Bern, Switzerland

**Keywords:** Trials, Recruitment, Methodological approach, Patient experience

## Abstract

**Background:**

Evidence-based establishment and implementation of best principles, laws and ordinances that regulate clinical research depend on the consultation and involvement of trial participants. Yet, guidance on methodological approaches to obtain trial participants’ perspectives is currently missing. This scoping review therefore aimed at identifying, describing and evaluating research approaches to obtain trial participants’ feedback on their views and experiences.

**Methods:**

We searched the electronic databases Medline and PsycInfo via Ovid and the Web of Science Core Collection. Clinical trials were included that involved adult participants that were conducted in selected high-income countries and that were published in peer-reviewed journals between 1985 and 2018. In addition, 29 expert interviews were conducted between March and May 2019.

**Results:**

Out of 5994 identified records, 23 articles were included in this review. Twelve studies used a qualitative approach, 10 were quantitative and one study used a mixed-method design. More than 75% of all work was conducted in the USA and the UK. The scoping review and the expert interviews highlighted that recruitment of participants was generally done through direct contact by principal investigators and/or study nurses or through searches in de-identified patient databases. Authors used surveys, interviews or focus group discussions. The tools used were either based on existing validated ones or developed and verified de novo with the support of experts and/or patient representatives.

**Conclusions:**

To our knowledge, this is the first methodological literature review of approaches to researching experiences of clinical trial participants where findings were triangulated with expert interviews. Covering a range of indications, trial phases and study settings, it demonstrates that clinical trial participant perspectives and experience is heavily under-researched. This casts doubt on the overall robustness of available insight into trial participants’ views and experiences. Our results demonstrate that the methodology for studying participant opinion, perception and experience should be adapted to the measure of interest and conform to the study population. Using valid patient experience data is the basis to evaluate existing legal and regulatory human subject research frameworks for their appropriateness from a patient perspective. Such an evaluation will be critical to empower research participants.

## Background

Thanks to human research, significant progress has been—and continues to be—made in medicine. However, research on humans is often accompanied by risks for those individuals subjected to the research. Therefore, the intervention into the physical and psychological integrity of study participants requires a regulatory frame based on ethical and legal norms. Such frameworks should ensure that the trial participants’ safety, well-being and rights are protected without jeopardising the validity and transparency of the trial and the production of high-quality data. They should be evidence-based and incorporate the attitudes and views from trial participants so that their expectations with respect to their protection and mitigation of risks associated with trial participation are considered.

Many of the clinical trial ordinances, guidelines and principles on clinical trial conduct indeed undergo continuous revision and refinement in order to adapt them to the changing research and cultural environment. Commonly, expert committees and stakeholders in clinical research convene to discuss the necessity for any revisions. However, even though some of those hearings are public, the views and concerns of prospective or former trial participants on specific aspects of trial participation are not commonly documented and integrated. Yet, the evaluation of the legal and ethical aspects of trial conduct through trial participants’ experiences, perspectives and preferences and feeding this information back into the legal, regulatory and ethical framework is essential in order to progress from a rather passive role of trial participants to one that is more empowering and participatory and leverages their views and experiences. Post-study follow-up research on patients’ perspectives consequently seeks to identify the underlying evidence and rationale as well as enabling factors for putting in place adequate guiding principles and legal frameworks. Such research is intended to cover the population in entire areas of jurisdiction as well as a broad range of types of trials and therefore requires an objective and broad representation of participants’ experiences and opinions.

Despite the importance of these validations, very little research in this direction has been conducted and there is no common understanding about a suitable approach and methodology for obtaining the trial participant’s perspective. Obstacles to such research may chiefly consist of data protection regulations that limit access to an extended study population; however, less obvious obstacles may also apply. In order to advance follow-up research on trial participants’ experiences and opinions, it is critical to understand and address these obstacles.

Over the past years, a considerable amount of studies has been conducted to evaluate the application of Good Clinical Practice (GCP) or legal frameworks pertaining to the conduct of clinical research. Such studies mainly addressed the aspects of health literacy, adequacy, reception and comprehension of the informed consent form [1–4] as well as expert opinions on confidentiality and data sharing [5, 6]. However, these studies did not necessarily involve the research participants. Other studies [7–9] looked at issues related to enrolment and retention of patients in clinical studies with the aim to understand the reasons and motivations for trial participation and ultimately to make clinical trials more effective [10, 11]. Recently, Planner and colleagues [12] conducted a scoping review to assess standardised measures of participants’ experiences in a clinical trial. They advocate for routine measurements of participants’ experiences within trials as a necessary aspect of quality improvement of trials and continuous increase of patient engagement in research.

The aim of the current study was to establish an overview of the different methodological approaches used to gather research participants’ views on their previous participation in a clinical trial and to review the various potential sources of bias in this sort of research. The study was mandated by the Swiss Federal Office of Public Health and timed with an ongoing evaluation of the Swiss Federal Act on Research involving Human Beings with the aim to develop a methodology to include participants’ perspectives in the evaluation of the implementation of the Act.

Our objectives were to:

- Identify clinical trials (involving any type of adult participant, intervention, comparison or outcomes) soliciting patient experience of trial participation;
- Characterise the methodology applied to approach trial participants and systematically describe the recruitment process, the development of research instruments and their administration;
- Obtain stakeholders’ feedback on the identified methodologies and their feasibility in the Swiss context; and
- Make recommendations for approaching trial participants for conducting research on their experience of trial participation.

### Review questions

This scoping review of published research articles was guided by the following two research questions:

- What methodological approaches are commonly used to identify and get in contact with persons who participated in clinical trials and to get their feedback?
- Does this research discuss the methodological challenges, notably the biases that these approaches (might) imply?

## Methods

For this scoping review, we used methodological approaches as previously described, following guidelines from the Preferred Reporting Items for Systematic Reviews and Meta Analyses extension for scoping reviews [13–17]. We conducted the review as a form of knowledge synthesis to address our exploratory research question by mapping key methodological approaches on participant experience of taking part in clinical trials. We systematically searched, selected and synthesised existing knowledge in the field.

### Search strategy

Two information specialists (HE and CA-H) developed the search strategy. Initially, topical seed records were collected non-systematically through web-searches and the PubMed similar article function. Guided by these seed records, a search strategy was designed in PubMed that was based on text words (synonyms and word variations) and database-specific subject headings for clinical trials, participants, qualitative assessment methods and perceived outcomes. The search retrieved 33/34 Medline-indexed seed papers. Using this search strategy, we searched the electronic databases Medline and PsycInfo via Ovid and the Web of Science Core Collection (last search December 18, 2018; Appendix 1). Retrieved records were exported to EndNote X8 and deduplicated.

To identify possible additional studies that escaped our electronic database searches, we conducted backward and forward citation chasing (i.e. we screened the bibliographic references and citations) based on all articles that were subjected to full-text screening. Cited-by lists were retrieved on March 14, 2019, from scopus.com or from the Web of Science. Likewise, selected seed records that were not retrieved by our database searches for various reasons were added as additional records identified through other sources.

### Inclusion and exclusion criteria

The scoping review considered studies based on the (i) types of participants (clinical trial participants); (ii) the concept (the methodological approach); (iii) the context (similar policy setting in health care research); and (iv) types of studies/publications. Furthermore, only studies published in English, German and French were considered for inclusion.

(i) *Types of participants*: This scoping review included only studies that researched adult participants in clinical trials.
(ii) *Concept*: The core concept examined was on the applied methodology of studies targeting clinical trial participants for a follow-up study (e.g. recruitment process, development of research instruments and their administration). In addition, the studies had to focus on patient experience of trial participation.
(iii) *Context*: This scoping review considered studies that were conducted in health care research settings similar to Switzerland, such as the member countries of the Organisation for Economic Co-operation and Development (OECD), outlined in the OECD Recommendation on the Governance of Clinical Trials (e.g. Australia, Canada, Europe, Japan, South Africa, USA and New Zealand) [18].
(iv) *Types of studies/publications*: This scoping review considered only primary research studies, which were published in peer-reviewed journals between 1985 and 2018, including qualitative, quantitative and mixed-method study designs.

Studies which reported on participant experiences of clinical trials in relation to Malaria, Tuberculosis, HIV and AIDS, Ebola, Neglected Tropical Diseases or mental health were excluded from the review, because of their too specific focus, and disease orientation when discussing trial participation.

### Definitions

For reasons of consistency, we used the following characteristics for distinguishing quantitative, qualitative and mixed-method research [19].

Quantitative research employs strategies of inquiry such as experimental and surveys, and collects statistical data on predetermined instruments. The author teams of the articles we analysed did not differentiate between quantitative research tools, such as surveys and questionnaires, but rather used them interchangeably.

Qualitative research is a holistic approach that involves discovery of certain phenomenon from the participant’s viewpoint. Most common research tools are interviews, focus group discussions and structured observation.

Mixed-method research is the collection and analysis of both, qualitative and quantitative data, the mixing of the two forms of data and their organisation into a specific research design.

### Study screening and selection

Two reviewers (JG, AS) screened the references based on their titles and abstracts. Potentially relevant references were retrieved in full-text and independently assessed by two reviewers based on pre-defined inclusion criteria (JG, AS). Any disagreements over eligibility were resolved by consensus.

### Data extraction

Data was extracted from the articles included in the scoping review by at least two independent reviewers (JG, AS) using the data extraction tool listed in Appendix 2. Data relevant to participant population, concept, context and the study methods significant to the scoping review question and specific objective was extracted. Any disagreements were resolved through discussion or with a third reviewer (JS). The data extraction tool was modified and revised in an iterative process during data extraction.

### Expert interviews

Simultaneously to the literature analysis, we conducted 29 expert interviews between March and May 2019. The expert interviews were chosen to complement and contextualise the findings of the scoping review with a special focus on the clinical trial landscape in Switzerland.

The expert interviews were semi-structured interviews based on an interview guideline of 10 questions and addressed the following themes: (a) references to follow-up studies on clinical trial participants; (b) opinions on different methodological approaches and their anticipated biases of conducting such studies; (c) ethical and data protection aspects in accessing participants of clinical trials; (d) suggestions of appropriate research tools; and (d) the potential willingness of the expert to facilitate and support a follow-up study on clinical trial participants.

The author team (JG, AS, CB) and the contractor (MG) of this study jointly identified the relevant experts in Switzerland in the three main language regions (French, Italian and German).

The telephone interviews’ duration averaged around 30 min and were audio and type recorded and analysed using framework analysis [20]. Data collection of the scoping review and the expert interviews were done independently; however, they were combined during data analysis and interpretation. In the “Discussion” section, findings from the expert interviews were triangulated with the findings from the scoping review by seeking convergence and corroboration.

## Results

### Basic features of the dataset

#### Expert interviews

Experts had diverse backgrounds being members of clinical trial units (*n* = 7), ethic review boards (*n* = 7) or interest groups (*n* = 6), industrial sponsors (*n* = 5) or researchers (*n* = 4). Experts were identified according to their position level without particularly focussing on gender balance. Overall, 12 women and 17 men were interviewed.

#### Scoping review

**Fig. 1:**
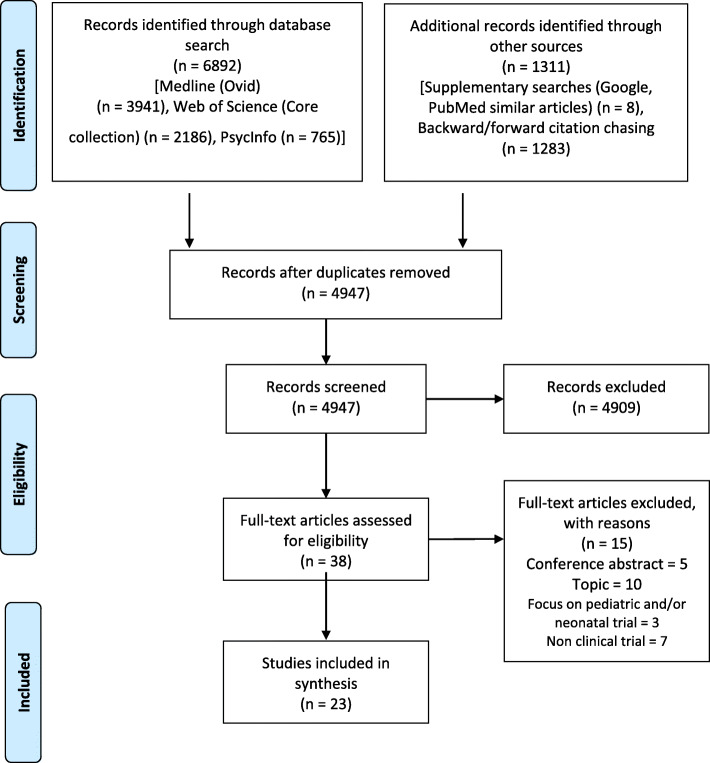
Scoping review study selection flowchart

Our electronic searches identified 6892 records and 1311 potentially eligible additional records were found through other sources (Fig. 1). A total of 4947 abstracts and titles were screened, and 38 articles were assessed on full-text level resulting in the exclusion of another 15 articles. Of the remaining 23 articles, only three were published before 2000, 15 after 2010 and 8 after 2015. Most studies were conducted in the USA (*n* = 12) and the UK (*n* = 5). Other settings were Australia (*n* = 3), Canada (*n* = 3), Portugal (*n* = 1) and Switzerland (*n* = 1); the country specifications for one international study were missing (Table 1).

**Table 1:** Summary of articles from 1985 to 2018 reporting on clinical trial experience of adult participants in countries and areas with health care settings similar to Switzerland

Study	Geographical location(s)	Number of trials or facilities in the study	Items	Number of participants (response rate)	Study design	Tool
Almeida et al. (2007) [21]	Portugal	Multiple studies, 1 facility	14	136 (100%)	Quantitative	Questionnaire
Au et al. (2015) [22]	Australia	14 trials, 1 facility	37	80 (96%)	Quantitative	Survey
Cox (2000) [23]	UK	Multiple trials	Not reported	55 (NA)	Qualitative	Interviews
DasMahapatra et al. (2017) [24]	International	PatientsLikeMe platform	12	1621 (24%)	Quantitative	Survey
Dayer et al. (2017) [25]	Switzerland	1 trial, 1 facility	24	103 (90%)	Quantitative	Survey
Harrop et al. (2016) [26]	UK	1 trial, multicentre	NA	10 (NA)	Qualitative	Interviews
Henzlova et al. (1994) [27]	USA & Canada	1 trial, 83 hospitals	10	3522 (74%)	Quantitative	Questionnaire
Johnson et al. (2008) [28]	UK	1 trial, 89 facilities	Not reported	1431 (37%)	Quantitative	Questionnaire
Kost et al. (2011) [29]	USA	Multiple trials, 10 centres	NA	96 (NA)	Qualitative	FGDs
Kost et al. (2014) [30]	USA	Multiple research projects; 15 centres	77	4961/18890 (26%)	Quantitative	Survey
Kvale et al. (2010) [31]	USA	1 trial, 1 facility	Not reported	4 (NA)	Qualitative	Interview
Lawton et al. (2003) [32]	USA	1 trial, 23 facilities		10 (NA)	Qualitative	Interview
Locock & Smith (2011) [33]	UK	Multiple trials	NA	42 (NA)	Qualitative	Interview
Mathieu et al. (2012) [34]	International	1 trial, multifacility	15 for long and 5 for short version	416 (33.4%)	Quantitative	Survey
Mattson et al. (1985) [35]	USA	2 multicentre trials,	Not reported	1202 (80%) for questionnaire 380 (95%) for interview	Mixed-methods	Interviews & questionnaires
Mello et al. (2018) [36]	USA	Multiple trials, 3 facilities	30	771 (79%)	Quantitative	Survey
Pflugeisen et al. (2016) [37]	USA	Multiple trials, 1 institute	45	90 (41%)	Quantitative	Survey
Pope et al. (2003) [38]	Canada	14 trials, 1 facility	39	190 (75%)	Quantitative	Survey
Tutton et al. (2018) [39]	UK	1 trial	NA	20	Qualitative	Interviews
Wootten et al. (2011) [40]	Australia	5 trials	NA	14 (of 30 deemed eligible)	Qualitative	FGDs
Yessis et al. (2012) [41]	USA	Multiple research projects; 15 centres	76	4961 (26%)	Quantitative	Survey
Yoder et al. (1997) [42]	USA	1 trial, 1 facility	23 for initial interview and 12 questions for exit interview	37	Qualitative	Interviews
Zaharoff & Cipra (2018) [43]	USA	Multiple trials, 1 facility	NA	21	Qualitative	FGDs

Drugs were the most frequent investigational medicinal product (IMP) across the studies assessed (*n* = 11) , surgical and behavioural interventions were investigated by two studies each and seven studies did not specify the intervention. Cancer was the most frequently investigated indication (*n* = 8), followed by cardiovascular diseases (*n* = 4) and chronic diseases (*n* = 3) , including type 2 diabetes, rheumatologic disease, Parkinson’s disease and multiple sclerosis. Five studies abstained from specifying any indication. Most cancer-related studies used qualitative methods (7/8) and were phase I clinical trials (6/8), while most or all studies assessing cardiovascular diseases used quantitative tools (3/4) and were phase III clinical trials (4/4).

The most frequent experimental frame was phase I clinical trials (*n* = 9), followed by phase III (*n* = 7) and phase II clinical trials (*n* = 6).

Some of the identified studies for this scoping review are interlinked, for example Kost et al. [29], [30] and Yessis [41] are iterative studies of a research programme.

Twelve studies used a quantitative research approach, ten studies used qualitative research tools and one study used both qualitative and quantitative research instruments as a mixed method approach. Basic features of included studies are summarised in Table 1.

### Development of research instruments

#### Quantitative tools

Five of the 12 quantitative reports did not provide any information on tool development [25, 27, 28, 34, 38], while Pflugeisen and Almeida used either established tools or modified existing tools [21, 37] (Table 2). Almeida et al. established a 14-item questionnaire derived from the questionnaires used by Bigorra and Baños and Van Gelderen et al. [44, 45]. Participants also completed the NEO Personality Inventory (NEOPI-R) consisting of 240 statements [46]. Pflugeisen et al. used a combination of approaches including a review of the literature and available surveys (i.e. third-party vendor, two internal departments and free online surveys) and two brainstorming sessions to design a satisfaction survey [37]. Also Au et al. relied on the available literature for the development of the questionnaire guided by the evidence-base for selection of rating scales [22]. Yessis, Kost and Mello and their colleagues consulted expert opinions using FGDs [30, 36, 41]. While Kost et al. identified themes through FGDs with research participants and research professionals, Mello et al. developed their survey based on FGDs, expert consultations and community advisory boards. DasMahapatra et al. was the only author team that relied on no existing tools or evidence and used an iterative tool development process [24].

**Table 2:** Summary of approaches to tool development

Approach used	Details
**Quantitative studies**	
Use / adaptation of established/validated tools	Almeida, 2007 [21] - Questionnaire based on the ones used by Bigorra and Baños and Van Gelderen et al. [44, 45] - Use of NEO Personality Inventory (NEOPI-R), validated for the Portuguese population Pflugeisen, 2016 [37] - Review of available surveys (third-party vendor, internal departments, free online surveys)
Literature review, informed by previous studies	Au, 2015 [22] - Literature review of previous surveys - Satisfaction rating scales guided by evidence-base Pflugeisen, 2016 [37] - No further details provided
Informed by expert opinions, focus group discussions, etc.	Kost, 2014 [30] - Survey themes based on FGDs with research participants and research professionals Mello, 2018 [36] - Questionnaire based on FGDs, consultation with experts and community advisory boards Pflugeisen, 2016 [37] - Brainstorming sessions with leadership team and research oversight committee of research institute Yessis, 2012 [41] - Questionnaire based on FGDs with research participants and professionals
Iterative development by researcher	DasMahapatra, 2017 [24] - No further details provided
No information provided	Dayer, 2017 [25] Henzlova, 1994 [27] Johnson, 2008 [28] Mathieu, 2012 [34] Pope, 2003 [38]
**Qualitative studies**	
Use / adaptation of established/validated tools	Cox, 2000 [23] - Semi-structured interview supplemented with previously validated quality of life (QOL) questionnaires (EORTC QLQ C-30 and Hospital Anxiety and Depression Scale) Kost, 2011 [29] - Modified the general hospital survey around patients’ perceptions of hospital care established by National Research Corporation (NRC) Picker to assess perceptions of research participants
Literature review, informed by previous studies	Harrop, 2016 [26] - Topic guide informed by findings of a previous sub-study Wootten et al., 2011 [40] - No further details provided
Informed by expert opinions, focus group discussions, etc.	Harrop, 2016 [26] - Topic guide informed by clinical experience of PI Zaharoff, 2018 [43] - FDGs facilitated by support and advocacy organisations Yoder, 1997 [42] - Interview guides based on themes informed by patients, reviewed by clinical experts (physicians, clinical oncology nurses)
Iterative development by researcher	–
No information provided	Kvale, 2010 [31] Lawton, 2003 [32] Locock, 2011 [33] Tutton, 2018 [39]
**Mixed-method study**	
No information provided	Mattson, 1985 [35]

Six of the quantitative studies were based on anonymous data collection [21, 22, 25, 30, 36, 37], while only Henzlova et al. used non-anonymous data collection to the best of our knowledge [27]. No information on anonymousness was available from DasMahapatra, Johnson, Mathieu and Pope and their colleagues [24, 28, 34, 38] (Table 2).

#### Qualitative tools

Four of the 10 qualitative studies did not offer any information on tool development [31–33, 39]. However, Cox stated to have used/adapted available tools [23]. Their structured interview was supplemented with two previously validated quality of life (QOL) questionnaires (EORTC QLQ C-30 and Hospital Anxiety and Depression Scale) [47, 48]. Kost et al. on the other hand, modified the general hospital survey established by National Research Corporation (NRC) Picker (a public institution focussing on healthcare consumer data) to incorporate new questions specifically addressing clinical research processes [29]. Wootten et al. developed a discussion outline based on information gained from the literature and the aims of the study [40]. Harrop et al.’s topic guide was informed by the findings of a previous sub-study [49] and also included the clinical experience of the principal investigator. The tool was further informed by their study research questions and methodological preference for an interview structure, which encourages participants to talk freely and develop their own stories about the trial and illness and treatment journeys more broadly. Zaharoff and Cipra approached support and advocacy organisations to present the concept of a FGD to their constituents [43]. Finally, the research team of Yoder et al. developed interview guides, which a panel of clinical experts, including physicians and clinical oncology nurses, reviewed for content validity [42]. The questions were derived from themes the healthcare team had heard from patients.

#### Mixed-method tools

No information is provided by Mattson et al. [35] on the research instrument development.

#### Expert interviews

For the development and validation of research instruments (questionnaire, interview guide, FGD guide), the experts recommended to include patient representatives and patient organisations in structured and moderated consultation processes.

### Recruitment of clinical trial participants

#### Quantitative studies

Of the 12 quantitative studies, six recruited survey participants solely via mail (email or postal, often not distinguished by the authors) [24, 25, 30, 34, 37, 38]. The response rate across those seven trials varied widely from 24% for DasMahapatra et al. [24] to 90% in the study from Dayer et al. [25] (Table 1). DasMahapatra, Dayer, Pope and their colleagues explicitly stated that there was no remuneration of participants, while the other four studies did not offer any details on participant incentives.

Seven studies established access to trial participants either through the clinical trial staff [22, 25, 27, 28, 34, 36] or the oversight committee of the clinical research institution [37]. Of these, two studies relied solely on the handing out of questionnaires and information onsite [22, 27], while in the trial of Johnson et al. health care professionals either posted a questionnaire together with a patient newsletter directly or distributed the material in the UK hospital clinics to surviving patients of the ‘Taxotere as Adjuvant ChemoTherapy’ trial [28]. Henzlova et al. represents the only study that embedded the survey into an ongoing long-term clinical trial on heart failure rather than designing a separate study. For this set of studies, varied response rates were observed with 96% for Au et al. [22], 74% for Henzlova et al. [27] and 37% for Johnson et al. [28] (Table 1). None of the studies reported on whether patients were remunerated. In a further study conducted by Mello et al., clinical trial Principal Investigators (PIs) chose from among three methods of survey delivery, i.e. email, regular mail, or in-person distribution in study clinic waiting rooms [36]. Four PIs chose regular mail, four chose the clinic and one used both. The response rate was 79% and participants were remunerated. Dayer et al. approached adult volunteers who took part in a first-in-human trial testing an experimental Ebola vaccine by sending an anonymous, internet-based satisfaction survey by email to all participants upon their completion of the 1-year trial [25]. Mathieu et al. sent invitations to the registered email addresses of participants who completed the ‘Stretching Trial’ [34]. Embedded within this invitation was a unique link that provided participants with password-protected access to the online survey. In the study by Yessis et al., the method of recruitment for fielding the survey instrument consisted of establishing a master dataset of all adult research participants who had enrolled in one or more research studies within the prior 2 years at the participating institution [41]. A random sample was selected from that dataset and the names and addresses of the selected individuals securely transferred to a commercial developer and vendor of health care surveys under the protection of appropriate confidentiality agreements.

Finally, Pflugeisen et al. mailed the survey to patients that were identified by the MultiCare Institute for Research and Innovation Research leadership team and the MultiCare Oversight Committee based on the following conditions: (i) having consented to a trial in the past year; or (ii) having completed a trial in the past year to which they had consented more than 1 year prior as target survey recipients [37].

One study used an independent platform to recruit trial participants: DasMahapatra et al. applied unrestricted convenience sampling of members of the patient-powered research network ‘PatientsLikeMe’, sending invitations via private electronic messages [24].

For four out of 12 studies, no detailed information on participant recruitment was provided [21, 30, 35, 38]. Kost et al. distributed a survey to 18,890 research participants at 15 NIH-supported clinical research centres [30], while Pope et al. mailed a written questionnaire to 253 subjects who had previously participated or were participating at the time of the survey in a randomised controlled trial (RCT) and who (i) had received a letter of information; (ii) had signed an informed consent document; and (iii) were involved in an RCT less than 5 years ago. Eligible participants of the trials were identified through chart review, newspaper ads, screened by telephone or upon visit to the clinic and interested participants were invited for a screening visit [38].

#### Qualitative studies

Most articles (8/10) except the ones by Tutton et al. [39] and Yoder et al. offered some insight into the recruitment strategy for the interviews and FGDs. While none of those studies explicitly stated that the participants were directly approached by the authors, in half of the studies the clinical trial staff made the initial contact with the trial participants for the reported studies [26, 29, 31, 33, 40]. In the work done by Harrop et al., eligible patients of the FRAGMATIC trial on advanced lung cancer were approached by the study nurse allowing interviewing almost the whole trial patient group except for one patient whose health had deteriorated [26]. In the study of Kvale et al., it was also a study nurse that screened and identified patients that were perceived physically and emotionally strong enough for participation in interviews [31]. This was done in a one-time, in-depth, face-to-face interview session resulting in a subjective judgement, based on the clinical experience and knowledge of the patient. The study nurse approached the patient, briefed them on the study and informed consent. A copy of the consent document was taken home by the patients for consideration. Only after the patient had consented to participate did the research group make contact for interview appointments. For the FGDs conducted by Wootten et al., participants were contacted by research nurses after trial involvement [40], while the recruitment selection for participation in an FGD in Kost et al. was guided by research coordinators who identified participants whom they judged likely to contribute actively to focus groups [29]. While all the mentioned studies relied on trial staff for access to trial participants, Locock and Smith followed a multi-pronged approach, in that recruitment was conducted through general practitioners, research networks, the Medical Research Council Clinical Trials Unit, patient and public involvement groups, trial clinics and researchers, newsletters and websites [33]. Purposive maximum variation sampling was used to recruit respondents with as wide a range of experiences and backgrounds as possible, including some who had declined to take part in a trial, had failed to meet eligibility criteria, or had withdrawn early.

In four of 10 studies, an external expert or organisation was involved in recruiting trial participants [23, 32, 33, 43]. In the study by Cox, for instance, patients that were offered participation in a phase I or II anti-cancer drug trial were invited to take part in an interview study of their trial experiences [23]. The permission to approach the patients for the interviews was given by the consultant oncologist [26, 31]. In a study conducted by Lawton et al., all patients scheduled to attend an annual post-study monitoring appointment were sent information about the study together with an invitation to be interviewed by an independent researcher. Patients were informed that participation was strictly voluntary and that all information shared would be treated in confidence [32]. The recruitment in Zaharoff and Cipra was organised with the support and advocacy organisations Cancer Support Community, SHARE, FORCE and the National Ovarian Cancer Coalition [43, 50–53].

Zaharoff and Cipra were the only authors among the qualitative studies assessed that offered information on whether patients were remunerated (they were). Except for Tutton et al., all studies were separate rather than embedded within a given clinical trial [39].

#### Mixed-method study

In the mixed-method study of Mattson et al. dating back to 1985, questionnaires were mailed to a 50% random sample of living participants of the ‘Beta-Blocker Heart Attack Trial’ regardless of whether they were currently active in the trial or not. In addition, they conducted interviews with ca. 10% of living participants of the ‘Aspirin Myocardial Infarction Study’ using a random sample close to the study end. Interviews were conducted in the patients’ homes by trained interviewers not previously known to patients and not associated with either the clinics or the coordinating centre [35].

#### Expert interviews

The interviewed experts proposed three possible strategies for the recruitment of study participants of still ongoing trials, irrespective of the methodological approach of studies, i.e. recruiting via Principle Investigators (PI) of clinical trials, through patient organisations and via social media channels.

The experts highlighted that recruiting via PIs, however, bears the risk of various biases; PIs may feel their implementation of the trial is being evaluated and only confident PIs may agree to collaborate. Similarly, some PIs may not want to participate because of additional time efforts (selection bias). Involving PIs may cause additional costs to the study in order to compensate for their efforts. Also, trial participants may feel uncomfortable revealing issues or negative experiences when their trial PI is involved (social desirability bias).

The recruitment via patient organisations was mentioned by some experts as another promising strategy, where for instance representatives of interest groups could actively involve patient representatives in the announcement and recruitment process.

A few experts mentioned recruitment via social media channels, notice boards in hospital waiting rooms or an internet announcement, yet experts did not consider the success rates of such a recruitment to be particularly high.

### Study administration

The scoping review identified different approaches of study administration used for the qualitative, quantitative and mixed-method studies. How research tools were used and their mode of delivery is shown in Table 3.

**Table 3:** Summary of study administration

Tool used, mode of delivery and administration	Quantitative studies (*n* = 12)	Qualitative studies (*n* = 10)	Mixed-method studies (*n* = 1)
**Research tool used**			
Self-administered internet-based survey	3 [24] [25] [34]		
Self-administered paper based questionnaires	6 [28] [30] [36] [37] [38] [41]		1 [35]
Questionnaire, but no information	3 [21] [22] [27]		
Face-to-face individual interviews		7 [23] [26] [31] [32] [33] [39] [42]	1 [35]
Focus group discussions		3 [29] [40] [43]	
**Mode of delivery and administration**			
On site (waiting room, meeting room)			
Questionnaire	3 [22] [27] [28]		
Face-to-face interviews		3 [23] [31] [39]	
Focus group discussion		1 [29]	
At home			
Questionnaire (email, postal delivery)	6 [24] [25] [30] [34] [37] [38]		1 [35]
Face-to-face interviews		2 [26] [33]	1 [35]
Mixed (on site and at home)	1 [36]	1 [23]	
No further information	2 [21] [41]	3 [40] [42] [43]	

#### Expert interviews

For a quantitative study design, a combination of online survey (use of own device) and paper questionnaire was proposed as the most promising method to reach the widest possible target group and to address all age groups. Based on the experience of several experts, the response rate was greater for patients who could independently enter their data electronically at home. Experts furthermore suggested that patients could use their waiting time in the hospital sensibly during one of the study visits, in line with the principle of voluntary participation, in order to fill out a questionnaire. Such an approach would have to be supported by the PIs. In order to ensure a good response for a questionnaire, for example, a voucher was proposed as an incentive, whereby a position paper of the joint ethics committees was mentioned as guideline for compensating study participants.

Other experts believed that face-to-face interviews were the better way to gain patient experience from clinical trials. At the same time, it was emphasised that a qualitative study design would be more expensive and more complex than carrying out a study via questionnaire.

### Time of data collection

For the quantitative studies, data collection was done while participants were still enrolled in the trial (*n* = 3), after the closure of the trial (*n* = 5), or both, in still ongoing and recently closed trials (*n* = 2). For two studies, the details were missing.

For the qualitative studies, data collection was done retrospectively after the closure of the trial (*n* = 5), or while participants where still enrolled in the trial (*n* = 2). Three author teams applied a prospective longitudinal approach to data collection to document participants’ views and perceptions in real time during different stages of the trial.

For the mixed-method study, data collection was done retrospectively after the closure of the trial.

Both, prospective and retrospective study designs to evaluate trial experience were suggested by the experts. For a prospective study design, no additional informed consent process would be needed but the limitation would be a much longer study duration, compared to a retrospective design. Within a prospective study, trial experience could be investigated over time with repeated data collection points, which was mentioned to be important by several experts, as experience of trial participation is closely linked to the changing health conditions of patients, duration and temporal associations of trial procedures.

A retrospective study approach was generally considered to be a good approach to investigate trial participants’ experiences; however, access to participants was identified as bottleneck, especially under the premises of data protection. The experts noticed that such an approach also bears an increasing risk of recall bias the further back in time the assessed trial experience lies. In addition, the experts highlighted the difficulty in accessing participant data, once trials are already closed.

### Themes measured

#### Quantitative studies

The quantitative studies generally measured broad themes relating to the informed consent process, motivation of trial participation, experience (e.g. the satisfaction with participation and trial conduct) and the willingness to participate again in a clinical trial and/or recommend participation to others (Table 4). Some research groups assessed more specific aspects related to conducting clinical trial research. Mello et al. for example was interested in the perception of the risk of data sharing [36].

**Table 4:** Summary of measured themes

Theme(s)	Details		
	Quantitative studies	Qualitative studies	Mixed-method study
Global measures of experience (e.g. satisfaction with participation)	Almeida, 2007 [21] - Attitudes of healthy volunteers Au, 2015 [22] - Satisfaction with participation Dayer, 2017 [25] - Satisfaction with participation Henzlova, 1994 [27] - Satisfaction with participation - Negative experiences Kost, 2014 [30] - Overall experience Pflugeisen, 2016 [37] - Satisfaction with participation Pope, 2003 [38] - Satisfaction with participation		
Measures of specific aspects of the trial (e.g. informed consent)	Almeida, 2007 [21] - Perception of the informed consent procedure Johnson, 2008 [28] - Post-trial results sharing (including mode of reception) Kost, 2014 [30] - Understanding of the components of informed consent and other critical information Mello, 2018 [36] - Perception of risk of data sharing Pope, 2003 [38] - Satisfaction with the level of information received through the informed consent process Yessis, 2012 [41] - Perception of information and the informed consent procedure	Harrop, 2016 [26] - Understanding and acceptance of randomisation - Equipoise and acceptability of control and intervention arm - Trial information Kost, 2011 [29] - Satisfaction with informed consent process Locock, 2011 [33] - Information of trial participants - Feelings about randomisation, placebo and control groups - Withdrawing from trial - View on feedback of trial results Zaharoff, 2018 [43] - Participant recruitment	
Participants perception of the positive and/or negative aspects of participation	Almeida, 2007 [21] Au, 2015 [22] Henzlova, 1994 [27] Mathieu, 2012 [34]	Harrop, 2016 [26] Kost, 2011 [29] Kvale, 2010 [31] Lawton, 2003 [32] Tutton, 2018 [39] Wootten, 2011 [40] Yoder, 1997 [42]	Mattson, 1985 [35]
Willingness to participate again / recommend to others	Almeida, 2007 [21] Dayer, 2017 [25] Kost, 2014 [30] Yessis, 2012 [41]	Yoder, 1997 [42] Locock, 2011 [33]	Mattson, 1985 [35]
Reasons for participating	Almeida, 2007 [21] Au, 2015 [22] DasMahapatra, 2017 [24] Henzlova, 1994 [27] Kost, 2014 [30] Mathieu, 2012 [34] Pflugeisen, 2016 [37]	Harrop, 2016 [26] Kost, 2011 [29] Kvale, 2010 [31] Lawton, 2003 [32] Locock, 2011 [33] Yoder, 1997 [42] Zaharoff, 2018 [43]	Mattson, 1985 [35]
Other	DasMahapatra, 2017 [24] - Barriers to trial participation - Necessary infrastructure to engage patients in trial design - Overall attitudes towards trials Dayer, 2017 [25] - Impressions regarding adverse events Henzlova, 1994 [27] - Effect of the trial on health conscious behaviour Kost, 2014 [30] - Level of autonomy exercised - Feeling respected and valued by the research team Yessis, 2012 [41] - Coordination of care - Respect - Trust	Cox, 2000 [23] - Psychosocial impact of trial participation (ways of coping with what was happening to them, identify consequences of trial involvement) Kvale, 2010 [31] - Perception of supportive care needs - Role of hope Locock, 2011 [33] - Attitudes to clinical trials (need for conducting clinical trial research, motivations for taking part, understanding trial process and design, receiving feedback on trial results) - Communication with health professionals Tutton, 2018 [39] - Desire to be involved in research decision-making Wootten, 2011 [40] - Role of social/family support in participating in the clinical trial Yoder, 1997 [42] - Expectations Zaharoff, 2018 [43] - Barriers to participation	Mattson, 1985 [35] - Benefits from participation

#### Qualitative studies

The qualitative approaches more often assessed the participants’ perception of the positive and/or negative aspects of participation. Across all studies, the reasons for participation was the most frequently assessed measure (*n* = 15), followed by participants’ perception of the positive and/or negative aspects of participation (*n* = 12) and measures of specific aspects of the trial, such as informed consent (*n* = 10). Some research groups assessed more unique measures not commonly seen in other studies. DasMahapatra et al., for instance, assessed (i) the barriers to trial participation; (ii) how to build a systematic infrastructure to engage patients in trial design; and (iii) overall attitudes towards trials [24]. Dayer et al. looked at the impressions of patients regarding adverse events [25], while Henzlova et al. assessed trial effects on health conscious behaviour [27]. Locock and Smith described the attitudes to clinical trials (i.e. the need for conducting clinical research, motivations for taking part, understanding trial process and design, receiving feedback on trial results) [33], Tutton et al. assessed the desire to be involved in research decision-making [39], Yoder et al. looked at expectations [42] and Zaharoff and Cipra at assessed barriers to participation [43]

#### Mixed-method study

Mattson et al. were interested in the perceived advantages and disadvantages in participating in clinical trials.

## Discussion

### Methodological approaches for trial participant follow-up research relevant for the Swiss context

Our scoping review yielded a limited amount of studies that fulfilled our eligibility criteria. This was also reflected in Planner et al.’s report and our results corroborate their finding that measuring clinical trial participant experience is a yet under-researched topic [12]. Our hypothesis was that access to trial participants was the main bottleneck to any such research, particularly considering applicable data protection legislation.

The scoping review and the expert interviews identified various approaches in accessing trial participants. Authors of follow-up study reports adapted their recruitment strategy based on their main research interest, which is in line with the advice of the interviewed experts. That the recruitment of trial participants for a follow-up study is most feasible during still ongoing trials emerged as dominant in both the scoping review and the expert interviews. Rather than a direct contact by the researchers, about half of the studies, irrespective of design, relied on contacting trial participants via the clinical research team. Another approach to access trial participants may be the involvement of patient groups, as advised by the interviewed experts, or an external professional survey company. This would, however, require the transfer of participants’ names and addresses while respecting all applicable confidentiality regulations and putting in place respective agreements (like in Yessis et al.) [41]. Yet another option to ensure access to trial participants and a more systematic collection of their views and perceptions would be the generation of a central database of trial participants with voluntarily provided contact details for such evaluations. Provided data protection regulations are adhered to, such databases are an important step towards increased patient engagement in clinical research.

Both, the expert interviews and the scoping review highlighted that quantitative and qualitative research methods are suited to understand trial participant’s views and experiences. While qualitative methods are better used to obtain a deeper understanding of the patient perspective, quantitative methods are better suited to summarise the collected patient experience data.

Mattson et al. [35] in their mixed-method study approach, highlight the sequential approach of open-ended personal interviews first to generate hypotheses, e.g. for the development of a quantitative questionnaire. In addition, qualitative studies are also preferred to gather greater depth of findings deriving from quantitative studies, as a consecutive follow-up process.

The scoping review and the experts furthermore highlighted that interviews result in a very high response, but come at the expense of costs, considerable efforts by the field team and logistics. Such interviews also involve a big effort in coding and analysing the lengthy protocols or transcripts. Implementing quantitative questionnaires comes at a lower cost and consequently permits a much larger sample size. However, a major disadvantage of such questionnaires can be lower response rates and a consequent possibility of response bias.

For our scoping review, some of the reports lacked some procedural detail to allow a full understanding and a comprehensive assessment. Such missing detail included the approach to establish contact with trial participants, development of survey instruments and study logistics (including remuneration of participants). More often than not, the authors did not give any rationale for the choice of research instrument.

### Methodological challenges and biases

Most of the reviewed reports identified some limitations regarding their methodological approach. Many of them saw the main limitations of their studies in the small sample size and the limited generalisation, so the results may not be representative of other patients’ experiences. Regarding recruitment of study participants, handing out questionnaires or sending them by mail may bear the risk of a biased sample towards either satisfied or unsatisfied patients (non-response bias) as discussed by Au et al. [22]. Au also saw a potential undue influence of patients being handed the survey by research staff and the expectation to complete it on site. Most of the studies that we reviewed assessed participants’ experience after trial participation ended, which bears the risk of a recall bias. Particularly for trials involving severely ill patients, a high rate of attrition may be observed due to deterioration and death [26]; therefore, only experiences of those well enough to participate in the study are being captured. Some authors saw a limitation in the cross-sectional set-up of their study [22, 24]. Particularly when studying pre-trial expectations and link them to experiences made during the trial, a longitudinal prospective approach may be more appropriate. Furthermore, the involvement of people that decline participation in a trial may also yield important information depending on the research question [36].

For many of the reviewed reports, a critical appraisal of the potential biases introduced through the selection of the methodological approach was lacking. This was particularly the case for what we considered the main obstacle to such research, access to a representative set of trial participants in compliance with data protection. Overall, the limitations of the reviewed studies greatly reflect the recommendations for describing participant experience measures issued by Planner et al. [54].

### Strength and limitation of the review

To our knowledge, this is the first methodological review of the literature regarding approaches to researching experiences of clinical trial participants, and it covers a wide range of indications, trial phases and study settings. Our literature search for the scoping review was limited to peer-reviewed publications dating between 1985 and 2018 that report results from geographical regions with a healthcare and clinical research context that is similar to the one in Switzerland and other higher-income countries. While this represents a long timespan covering most of the recent developments and major innovations in guidelines and regulations pertaining to clinical research, our results may not be fully extrapolatable to other settings. Furthermore, we acknowledge that our review only includes studies conducted on adult trial participants, omitting an important group of particularly vulnerable patients, children and adolescents. Since this represents a quite distinct sub-population and requires a distinct methodological approach to assess their experiences (potentially including their caretakers’ experiences as well), we decided to focus on the adult trial population for this review.

We identified possible methodological obstacles to assess participants’ views by means of scoping review and expert interviews. As discussed in the updated JBI guidance [55, 56], the consultation of stakeholders is seen as an important part of the conduct of a scoping review, for various reasons: to refine the approach and to disseminate findings for example. Due to the timing of our mandate and the simultaneous conduct of the expert interviews and the analysis of the identified manuscripts, the expert opinions were in our case rather used to complement and contextualise the findings of the scoping review, than to refine the methodological approach of the literature study.

We did not contact study authors to ask for the missing methodological and procedural details. As another limitation pertaining to our search strategy, we increased specificity in some search blocks to scale down the enormous number of hits we would have obtained otherwise: For the concept on ‘patient participation’, we required text words to be present in the title only and we added the concept on ‘feedback’. We ensured that our PubMed search still retrieved 97% of the seed papers, i.e. eligible papers that were known before designing the search strategy. Another limitation might be the non-use of databases like CINAHL and Embase. Due to these limitations, we cannot provide any guarantee that we did not miss relevant methodological aspects.

This scoping review aimed to map the approaches applied to assess trial participant’s perspectives on their involvement in clinical trials. Our results show variability in the selection of quantitative, qualitative and mixed-methods, and the mode of study administration as well as in the themes of importance to participants that were investigated. Based on these findings, further research including expert and stakeholder consultation will be needed to develop guidance for the selection of the most appropriate methodology in a specific context.

## Conclusions

With our review, we provide an overview of the methodological approaches to research on experiences of clinical trial participants with the aim to advance research in this area. In contrast to the considerable amount of data available on participants’ views regarding their participation experiences, methodological approaches used to identify and get in contact with persons who participated in clinical trials were scarcely reported. With this report, we hope to contribute to advancing this important field of future research.

## Data Availability

All data generated or analysed during this study are included in this published article and its supplementary information files.

## Appendix 1

### Search strategy

#### Ovid Medline ALL (3941 hits, 2018-12-18)

(Trial.ti,ab. OR Trials.ti,ab. OR Health research.ti,ab. OR clinical research.ti,ab. OR exp Research Subjects/ OR research subject.ti,ab. OR research subjects.ti,ab. OR research participant.ti,ab. OR research participants.ti,ab.) AND (Participation.ti. OR participant*.ti. OR exp Patient Participation/ OR ((Patient.ti. OR Patients.ti. OR Subject .ti. OR Subjects.ti. OR trial.ti. OR trials.ti.) AND (Participated.ti. OR Participating.ti. OR Participate.ti. OR Partake.ti. OR Partaking.ti. OR Take part.ti. OR Taking part.ti. OR Took part.ti. OR Taken part.ti. OR Involved.ti.)) OR ((Patient.ti. OR Patients.ti.) AND (trial.ti. OR trials.ti.) AND (Questionnaire.ti. OR Questionnaires.ti. OR survey .ti. OR surveys.ti. OR surveyed.ti. OR focus-group.ti. OR focus-groups.ti. OR Interview.ti. OR Interviews.ti. OR Interviewed.ti. OR Teleconference.ti. OR Teleconferences.ti. OR qualitative.ti.))) AND (Questionnaire.ti,ab. OR Questionnaires.ti,ab. OR survey .ti,ab. OR surveys.ti,ab. OR surveyed.ti,ab. OR focus-group.ti,ab. OR focus-groups.ti,ab. OR Interview.ti,ab. OR Interviews.ti,ab. OR Interviewed.ti,ab. OR Teleconference.ti,ab. OR Teleconferences.ti,ab. OR qualitative assessment.ti,ab. OR Qualitative study.ti,ab. OR Qualitative studies.ti,ab. OR Qualitative Evaluation.ti,ab. OR Qualitative research.ti,ab. OR Randomized Response Technique.ti,ab. OR Randomized Response Techniques.ti,ab. OR Randomised Response Technique.ti,ab. OR Randomised Response Techniques.ti,ab. OR exp "Surveys and Questionnaires"/ OR exp Focus Groups/ OR exp Qualitative Research/ OR exp Interviews as Topic/ OR exp Evaluation Studies as Topic/ OR Evaluation Study.ti,ab. OR Evaluation Studies.ti,ab. OR Evaluation Studies.pt. OR exp Clinical Trials as Topic/) AND (Feedback.ti,ab. OR point of view.ti,ab. OR motivation.ti,ab. OR motivations.ti,ab. OR Expectation.ti,ab. OR Expectations.ti,ab. OR Attitude.ti,ab. OR Attitudes.ti,ab. OR Opinion.ti,ab. OR Opinions.ti,ab. OR Perception.ti,ab. OR Perceiv*.ti,ab. OR Perspectives.ti,ab. OR Prefer*.ti,ab. OR Experience.ti,ab. OR Experiences.ti,ab. OR Understanding.ti,ab. OR View.ti,ab. OR Views.ti,ab. OR Satisfaction.ti,ab. OR quality of life.ti,ab. OR exp Attitude to Health/ OR exp Patient Satisfaction/ OR exp Quality of Life/ OR exp Health Communication/)

#### PsycInfo (765 Hits, 2018-12-18)

(Trial.ti,ab. OR Trials.ti,ab. OR Health research.ti,ab. OR clinical research.ti,ab. OR exp Experimental Subjects/ OR research subject.ti,ab. OR research subjects.ti,ab. OR research participant.ti,ab. OR research participants.ti,ab.) AND (Participation.ti. OR participant*.ti. OR exp Client Participation/ OR ((Patient.ti. OR Patients.ti. OR Subject .ti. OR Subjects.ti. OR trial.ti. OR trials.ti.) AND (Participated.ti. OR Participating.ti. OR Participate.ti. OR Partake.ti. OR Partaking.ti. OR Take part.ti. OR Taking part.ti. OR Took part.ti. OR Taken part.ti. OR Involved.ti.)) OR ((Patient.ti. OR Patients.ti.) AND (trial.ti. OR trials.ti.) AND (Questionnaire.ti. OR Questionnaires.ti. OR survey .ti. OR surveys.ti. OR surveyed.ti. OR focus-group.ti. OR focus-groups.ti. OR Interview.ti. OR Interviews.ti. OR Interviewed.ti. OR Teleconference.ti. OR Teleconferences.ti. OR qualitative.ti.))) AND (Questionnaire.ti,ab. OR Questionnaires.ti,ab. OR survey .ti,ab. OR surveys.ti,ab. OR surveyed.ti,ab. OR focus-group.ti,ab. OR focus-groups.ti,ab. OR Interview.ti,ab. OR Interviews.ti,ab. OR Interviewed.ti,ab. OR Teleconference.ti,ab. OR Teleconferences.ti,ab. OR qualitative assessment.ti,ab. OR Qualitative study.ti,ab. OR Qualitative studies.ti,ab. OR Qualitative Evaluation.ti,ab. OR Qualitative research.ti,ab. OR Randomized Response Technique.ti,ab. OR Randomized Response Techniques.ti,ab. OR Randomised Response Technique.ti,ab. OR Randomised Response Techniques.ti,ab. OR exp Surveys/ OR exp Questionnaires/ OR exp Group Discussion/ OR exp Qualitative Research/ OR exp Interviews/ OR Evaluation Study.ti,ab. OR Evaluation Studies.ti,ab. OR exp Clinical Trials/) AND (Feedback.ti,ab. OR point of view.ti,ab. OR motivation.ti,ab. OR motivations.ti,ab. OR Expectation.ti,ab. OR Expectations.ti,ab. OR Attitude.ti,ab. OR Attitudes.ti,ab. OR Opinion.ti,ab. OR Opinions.ti,ab. OR Perception.ti,ab. OR Perceiv*.ti,ab. OR Perspectives.ti,ab. OR Prefer*.ti,ab. OR Experience.ti,ab. OR Experiences.ti,ab. OR Understanding.ti,ab. OR View.ti,ab. OR Views.ti,ab. OR Satisfaction.ti,ab. OR quality of life.ti,ab. OR exp Health Attitudes/ OR exp Client Satisfaction/ OR exp Quality of Life/)

#### Web of Science Core Collection (2186 Hits, 2018-12-18)

# 1 **TOPIC:** ((Trial OR Trials OR "Health research" OR "clinical research" OR "research subject" OR "research subjects" OR "research participant" OR "research participants") AND (Questionnaire OR Questionnaires OR survey OR surveys OR surveyed OR "focus group" OR "focus groups" OR Interview OR Interviews OR Interviewed OR Teleconference OR Teleconferences OR "qualitative assessment" OR "Qualitative study" OR "Qualitative studies" OR "Qualitative Evaluation" OR "Qualitative research" OR "Randomized Response Technique" OR "Randomized Response Techniques" OR "Randomised Response Technique" OR "Randomised Response Techniques" OR "Evaluation Study" OR "Evaluation Studies") AND (Feedback OR "point of view" OR motivation OR motivations OR Expectation OR Expectations OR Attitude OR Attitudes OR Opinion OR Opinions OR Perception OR Perceiv* OR Perspectives OR Prefer* OR Experience OR Experiences OR Understanding OR View OR Views OR Satisfaction OR "quality of life"))

Indexes = SCI-EXPANDED, SSCI, A&HCI, CPCI-S, CPCI-SSH, BKCI-S, BKCI-SSH, ESCI, CCR-EXPANDED, IC Timespan = All years

# 2**TITLE:** (Participation OR participant* OR ((Patient OR Patients OR Subject OR Subjects OR trial OR trials) AND (Participated OR Participating OR Participate OR Partake OR Partaking OR "Take part" OR "Taking part" OR "Took part" OR "Taken part" OR Involved)) OR ((Patient OR Patients) AND (trial OR trials) AND (Questionnaire OR Questionnaires OR survey OR surveys OR surveyed OR focus-group OR focus-groups OR Interview OR Interviews OR Interviewed OR Teleconference OR Teleconferences OR qualitative)))

Indexes = SCI-EXPANDED, SSCI, A&HCI, CPCI-S, CPCI-SSH, BKCI-S, BKCI-SSH, ESCI, CCR-EXPANDED, IC Timespan = All years

# 3 #1 AND #2

Indexes = SCI-EXPANDED, SSCI, A&HCI, CPCI-S, CPCI-SSH, BKCI-S, BKCI-SSH, ESCI, CCR-EXPANDED, IC Timespan = All years

## Appendix 2

### Data extraction items

**Table:**

**Study Details and Characteristics**	
Data extraction (name and date)	
Study citation details (e.g. author/s, date, title, journal, volume, issue, pages)	
Location/Countries	
Number of trials or facilities in the study	
Trial phase, indication, IMP	
**Details/Results extracted from study**	
Research objective	
Research method	
Research tools	
Tool details, items	
Method description	
Recruitment of trial participants	
Total number of participants	
Trial stage	
Remuneration	
Findings (main categories)	
Discussion (main categories)	
Reported Limitation of the methodological approach	
